# Human Endophthalmitis Caused By Pseudorabies Virus Infection, China, 2017

**DOI:** 10.3201/eid2406.171612

**Published:** 2018-06

**Authors:** Jing-Wen Ai, Shan-Shan Weng, Qi Cheng, Peng Cui, Yong-Jun Li, Hong-Long Wu, Yi-Min Zhu, Bin Xu, Wen-Hong Zhang

**Affiliations:** Huashan Hospital of Fudan University, Shanghai, China (J.-W. Ai, S.-S. Weng, Q. Cheng, P. Cui, Y.-M. Zhu, B. Xu, W.-H. Zhang);; BGI genomics, BGI-Shenzen, Shenzen, China (Y.-J. Li);; Binhai Genomics Institute, Translational Genomics Center, Tianjin, China (H.-L. Wu)

**Keywords:** Human endophthalmitis, pseudorabies, viruses, *Herpesviridae*, *Alphaherpesvirinae*, Suid herpesvirus 1, swineherder, hog farm, high-throughput nucleotide sequencing, endophthalmitis, Jiangxi Province, China

## Abstract

We report human endophthalmitis caused by pseudorabies virus infection after exposure to sewage on a hog farm in China. High-throughput sequencing and real-time PCR of vitreous humor showed pseudorabies virus sequences. This case showed that pseudorabies virus might infect humans after direct contact with contaminants.

Pseudorabies virus (PRV) primarily infects swine and has several secondary hosts, including cattle, dogs, and cats. PRV, also called Aujeszky disease virus or Suid herpesvirus 1, is a member of the *Alphaherpesvirinae* subfamily within the family *Herpesviridae*. PRV infection has not been confirmed in humans (*1*), but previous reports have suggested the possible presence of PRV infection in 3 immunocompetent humans in whom fever, sweating, and neurologic complaints developed; virus neutralization and immune precipitation tests were positive for PRV antibody (*2*,*3*). We report a human case of human infectious endophthalmitis caused by PRV in a woman from Jiangxi Province, China, in July 2017. 

## The Study

On June 14, 2017, sewage from a hog farm spilled onto a 46-year-old woman from Jiangxi Province, China, who worked as a swineherder; her daily work was to feed swine and clean hoggery sewage.. The next day, she had a headache and fever of 39.5°C. Three days later, she became visually impaired and was admitted to a local hospital, where she was treated empirically with meropenem, vancomycin, and acyclovir. On June 29, after no significant improvement, she was transferred to Huashan Hospital of Fudan University (Shanghai, China), to treat unresponsive fever, headaches, and visual impairment. 

**Figure 1:**
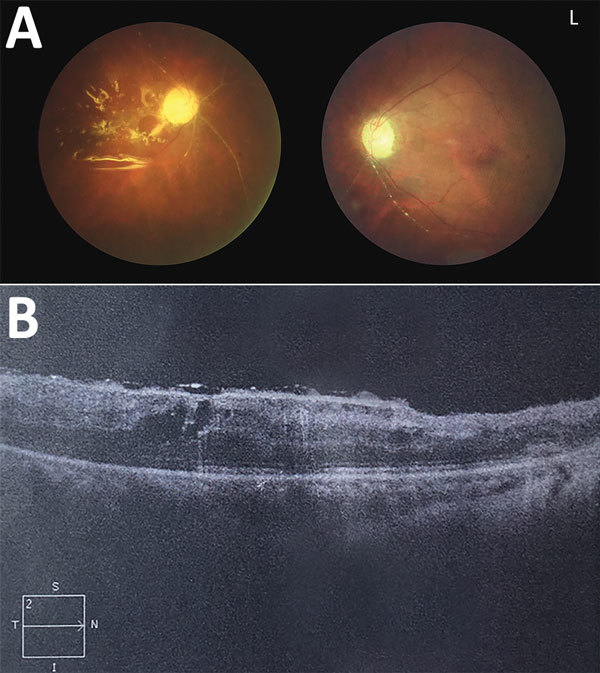
Ocular examination conducted 2 months after eye surgery in patient with human endophthalmitis caused by pseudorabies virus, China, 2017. A) Fundus photography of both eyes showing retinal necrosis and occlusive vasculitis and postoperative change after pars plana vitrectomy with silicone oil injection in the right eye. B) Optical coherence tomography of the patient’s right eye showed postoperative change after pars plana vitrectomy. 2, scan depth (2 mm); I, inferior, L, left eye; N, nasal side; S, superior; T, temporal side.

On examination, she had palpebral conjunctival congestion, and visual acuity to light perception of both eyes had worsened. Slit-lamp examination showed keratic precipitates and Tyndall effect flare. Funduscopic examination revealed vitreous opacity (Figure 1, panel A) and a pale white lesion on the posterior pole of the right eye (Figure 1, panel B), which suggested acute retinal necrosis and occlusive vasculitis. Results of routine laboratory testing were normal, including serology tests for HIV and hepatitis B and C, T-SPOT.*TB* test (Oxford Immunotec Ltd., Oxford, UK), blood culture, cryptococcal latex agglutination test, autoantibodies, and cerebrospinal fluid test. Test results for plasma Epstein-Barr virus and cytomegalovirus (CMV) IgG were positive; IgM was negative for both pathogens.

The on-staff ophthalmologist diagnosed endophthalmitis in the patient, considering viral infection as most likely. The patient was transferred to the ophthalmology department for vitrectomy surgery on the right eye on June 30. During the operation, ≈2 mL of vitreous humor was taken for culture and next-generation sequencing (NGS) (Technical Appendix)

**Figure 2:**
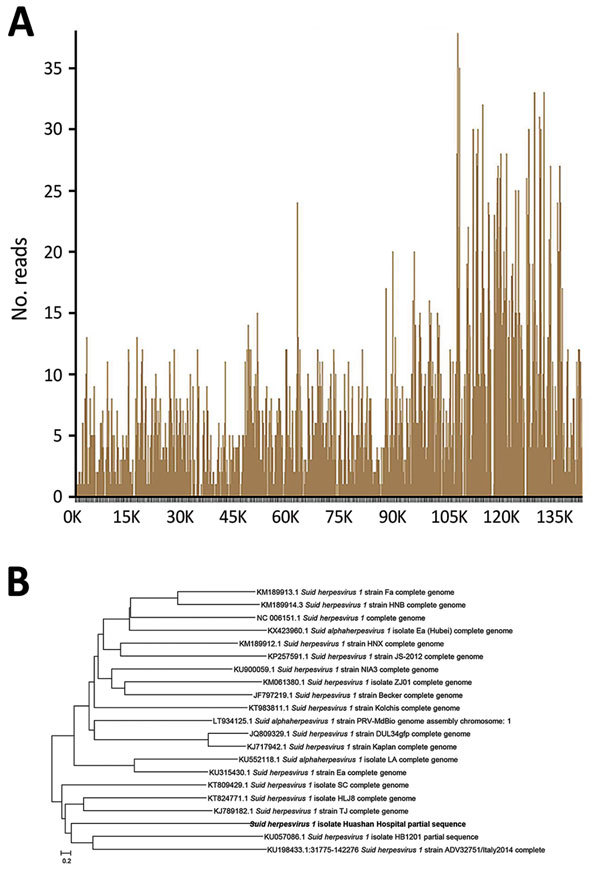
Results of gene sequencing, serologic testing, and phylogenetic analysis of pseudorabies virus from patient with human endophthalmitis, China, 2017. A) Sequencing of Suid herpesvirus 1 (pseudorabies virus) yielded a total coverage of 84%. B) Evolutionary relationships of taxa. Phylogenetic analysis disclosed a close connection between the isolate from the patient (boldface) and 4 other Suid herpesvirus 1 strains. Scale bar indicates amino acid substitutions per site.

On July 2, NGS results showed 4,832 unique sequence reads of PRV in vitreous humor, covering 84% of the nucleotide sequences (Table; Figure 2, panel A); NGS results for cerebrospinal fluid (CSF) were negative for PRV. Other detected sequences were within laboratory reference ranges. On the basis of NGS results, the physicians, who suspected that the patient may have acquired PRV infection, immediately initiated valacyclovir therapy. Sanger sequencing (Technical Appendix Figure 1) and PCR analysis (Technical Appendix Figures 2, 3) confirmed identification of PRV in vitreous humor (*4**–**6*). Phylogenetic analysis disclosed a close connection between the isolated strain and 3 emergent and highly pathogenic PRV variants in China (Figure 2, panel B; Technical Appendix) (*7*,*8*). 

**Table:** Pathogens detected by using next-generation sequencing in vitreous humor from a patient with human endophthalmitis caused by pseudorabies virus, China, 2017

Pathogen	Coverage, %	Depth, bp	Unique reads
Virus			
* Suid herpesvirus 1*	84	6.8	4,832
* Bovine herpesvirus 5*	8.9	1	1
Bacteria			
* Thermoanaerobacter wiegelii*	0.52	5.3	9
* Corynebacterium urealyticum*	0.56	1	0
* Haloquadratum walsbyi*	0.47	2.8	0
* Brachyspira pilosicoli*	0.41	1	1
* Candidatus *Nitrososphaera	0.46	1	0
Fungus			
* Cryptococcus gattii*	0.46	1	1

To further validate our results, we constructed a plasmid and a standard curve for real-time PCR and obtained quantitative results of viral DNA load as 2.7 × 10^6^ copies/mL (Technical Appendix Figure 3). To rule out contamination, we performed PCR of the PRV on a control group of 7 persons; all were negative (Technical Appendix Figure 2). Other possible viral causes of infective endophthalmitis, such as varicella-zoster virus, herpes simplex virus, and cytomegalovirus, were excluded through PCR. One week after surgery, culture result for vitreous humor was negative; the patient’s fever and headache had resolved, and visual acuity had improved slightly. The patient was discharged on July 11; during the last clinical check-up (December 6, 2017), the visual acuity of her left eye had improved to 0.2, and the right eye remained at slight light perception.

During the follow-up period, we obtained the patient’s plasma and CSF samples and ordered PRV antibody testing. PRV antibody was detected in all plasma samples at 4 and 5 months after disease onset and in CSF samples at 2 weeks to 2 months after disease onset, indicating the patient’s previous contact with PRV (Technical Appendix Figure 4). Epstein-Barr virus, CMV, and PRV serologic tests were also conducted on control samples and ruled out the possibility of cross-reaction (Technical Appendix Table 2) (*9**,**10*). Although the patient experienced headache and fever during disease progression and CSF PRV antibody test was positive, routine tests, NGS, and PCR of the CSF all failed to disclose abnormality. Therefore, we do not have enough evidence to suggest possible PRV central nervous system infection. Considering epidemiologic history, clinical symptoms, and serologic and molecular testing results, we diagnosed PRV endophthalmitis.

## Conclusions

The first 2 suspected cases of human PRV infection were reported in 1914, but detection of antibodies or cultivation of the virus had failed. In 1987, Mravak reported 3 suspected cases of human PRV infection with positive serum antibodies (*2*). All 3 patients were immunocompetent and their clinical manifestation occurred 1–3 weeks after possible animal contact. Initial symptoms included fever, sweating, and weakness; later, central nervous system symptoms developed. Some symptoms persisted for months, and serologic PRV antibodies were positive 5–15 months after the onset of clinical symptoms. In our study, the patient had similar symptoms, plus visual impairment and a unique infection route of PRV through direct exposure to contaminants.

Since 2011, prevalence of PRV infection among swineherds in China has risen several times (*11*). Among swineherds in Jiangxi Province, studies have shown varying positive rates for PRV DNA: from 5.5% to 26.5% during 2014, (*12*), and of positive PRV antibodies, from 84.4% to 89.9% during September 2013–September 2015 (*13*). PRV vaccine is still provided for swineherds on a voluntary basis rather than as a requirement in China, and swine were not vaccinated in the hoggery in which this patient worked at that time. On the day of disease onset, the patient’s eyes were directly contaminated with sewage containing pig excrement when cleaning pig sties. Although no previous study had reported a confirmed case of PRV-caused infectious endophthalmitis, the typical infectious route in this case made hoggery the most probable infectious source in this study.

NGS is marked by its rapid diagnostic ability to precisely identify certain pathogens in peripheral blood, respiratory, and CSF samples. In this case, NGS testing in facilitating the diagnosis of infective endophthalmitis is supported. The fact that real-time PCR and Sanger sequencing results were consistent with the NGS results further validated the credibility of this technique. Furthermore, a serologic test was conducted during the follow-up period; results showed that, even 5 months after symptom onset, the antibodies to PRV in plasma were still active, and the antibodies in CSF persisted during the entire follow-up period (Technical Appendix Figure 4). This result is similar to that reported in 1987, indicating that PRV antibodies may persist long after the initial infections. 

In summary, this case of PRV-caused human infectious endophthalmitis indicates that PRV could affect humans through direct contact with pig contaminants. NGS of the vitreous humor provided a strong technical support for rapid diagnosis of PRV infection in this patient. However, the pathogenesis of PRV infection remains to be explored. This case stresses the importance of mandatory PRV vaccine among swineherds and the necessity for workers in the breeding industry to increase awareness of self-protection when handling animal containments.

Technical Appendix Detailed descriptions of DNA analysis, antibody identification testing, and phylogenetic analysis of Suid herpesvirus.
