# Supplementary material for: Human Endophthalmitis Caused By Pseudorabies Virus Infection, China, 2017
Source: Emerg Infect Dis. 2018 Jun;24(6):1087–90. doi: 10.3201/eid2406.171612 (PMC6004832; doi:10.3201/eid2406.171612)
Supplement: Technical Appendix — Detailed descriptions of DNA analysis, antibody identification testing, and phylogenetic analysis of Suid herpesvirus. [file 17-1612-Techapp-s1.pdf]

Article DOI: https://doi.org/10.3201/eid2406.171612

Human Endophthalmitis Caused By Pseudorabies Virus Infection, China, 2017

Technical Appendix

Next Generation Sequencing

The ophthalmologist collected 2 mL of the vitreous humor from the patient according to standard procedures, and we processed it accordingly. A 1.5ml microcentrifuge tube with 0.5mL sample and a 1g, 0.5mm glass bead were attached to a horizontal platform on a vortex mixer and agitated vigorously at 2,800–3,200 rpm for 30 minutes. A 0.3mL sample was separated into a new 1.5 mL microcentrifuge tube and DNA was extracted by using the TIANAmp Micro DNA Kit (Tiangen Biotech, Beijing, China) according to the manufacturer's recommendation. We constructed DNA libraries through DNA-fragmentation, end-repair, add A-tailing, adaptor-ligation and PCR amplification. We used Agilent 2100 for quality control of the DNA libraries. Quality qualified libraries were sequenced by BGISEQ-100 platform.

Polymerase Chain Reaction, Sanger Sequencing and Real-time PCR

DNA was extracted by using the DNeasy Blood & Tissue Kit (QIAGEN, Hilden, Germany) for extraction according to the manufacturer's recommendations and finally eluted in 100µL of buffer. Three pairs of specific primers were designed according to related articles (*1*,*2*,*3*) and the sequences of primers are summarized in Technical Appendix Table 1. All oligonucleotides were synthesized by BGI.

All PCRs were completed by using the Takara RR902A Kit in a total reaction volume of 25 µL. For a single reaction, 8.5 µL RNase-free water, 12.5 µLPremix Taq and 2 µL primers were merged as a master mix. Finally, we added the 2 µL DNA template and PCR was completed by using a ProFlex PCR System. The following thermal profile was used: PCR initial activation step at 95°Cfor 5 min; 35 cycles of 3-step cycling consisting of denaturation at 95°C for 30 s, annealing at 55°C for 30 s, and extension at 72°C for 90 s; finally extension at 72°C for

5 min. After obtaining positive results by electrophoresis, Sanger sequencing was performed by BGI as of which result were shown in Technical Appendix Figure 1.

PCRs were conducted among vitreous humor, plasma and cerebrospinal fluid obtained during follow-ups and only in vitreous humor was the PCR result positive (Technical Appendix Figure 2A). Besides, we also conducted PCR of pseudorabies virus in specimens of other patients during the same period, and the results were all negative, ruling out the possibility of contamination (Technical Appendix Figure 2B).

Real-time PCR were used to measure the viral load. Primer PRV -gE-Sybr were used since it works best. We ligated the PCR product to the Peasy-T3 plasmid and constructed standard curves with eight different concentration gradients from 10^2 to 10^9. The same pair of primers and the same profile of cycling stage were used and the melt curve stage was 95°C for 15 s, 60°C for 60 s, followed by 95°C for 30 s and 60°C for 15 s at last. The quantitative result of the viral DNA load is $2.7*10^3$ copies/uL (File 3, Figure S3).

Serologic Tests

IDEXX PRV gB Antibody Test Kit (IDEXX Laboratories,Westbrook, ME, USA) was used to test the antibodies of the patient's plasma, vitreous humor and cerebrospinal fluid. Protein gB is a conserved protein of PRV and present in all kinds of pseudorabies virus. The principle of this kit is that the PRV gB antibodies in the sample will bind to the antigen coated in the microwell, competing with enzyme-conjugated monoclonal antibodies. If no PRV gB antibodies are present in the test sample, the conjugated antibodies are free to react with the antigen. In the presence if enzyme, the substrate is converted to a product that reacts with the chromophore to generate a blue color.

Samples diluted by sample diluent were added to a PRV antigen-coated plate. After incubation and elution, OD values of each microwell were evaluated by spectrophotometer at 650 nm. Results are calculated by dividing the A(650) of the sample by the mean A(650) of the negative control, resulting in a sample/negative (S/N) value. The quantity of antibodies to PRV is inversely proportional to the A(650) and, thus, to the S/N value. If the S/N value is no higher than 0.60, than the result is considered positive.

Serologic tests of EBV/CMV/HSV-1/HSV-2/VZV were conducted by KingMed Diagnostics (Shanghai) laboratory. Besides plasma collected from this patient, control samples used were plasma from the same 20 patients who have been the control group in PRV serologic test. EBV IgG, HSV-1&2 antibodies and VZV antibodies were tested through euzymelinked immunosorbent assay. CMV IgG were tested through electrochemiluminescence detection method.

The results were shown in Technical Appendix Table 2. Serologic tests of HSV 1/2 and VZV were conducted on this patient and the results were negative. As for EBV and CMV antibodies, IgG for both viruses were positive while IgM were both negative. In the control group, 19 of 20 patients had positive plasma EBV IgG and all 20 patients had positive plasma CMV IgG while their PRV antibodies were all negative, which ruled out the possibility of false-positive serologic test of PRV due to cross-reaction with EBV or CMV serologic tests. Besides, the serologic test results of EBV and CMV were in accordance with the global epidemiologic data: EBV infects more than 90% of the world's adult human population (*4*) while CMV seroprevalence rates ranging 40–100% of the adult population (*5*). Therefore, we believe that the above data will further support the PRV serologic tests results in our study.

Phylogenetic Analysis

We inferred the evolutionary history of the Suid herpesvirus by using the Neighbor-Joining method in making a phylogenetic tree (Main article, Figure 2B). The optimal tree with the sum of branch length = 171.41397980 is shown. The tree is drawn to scale, with branch lengths in the same units as those of the evolutionary distances used to infer the phylogenetic tree. The evolutionary distances were computed by using the Maximum Composite Likelihood method and are in the units of the number of base substitutions per site. The analysis involved 21 nt sequences. All positions containing gaps and missing data were eliminated. There were a total of 56899 positions in the final dataset. Evolutionary analyses were conducted in MEGA7. The scale bar represents 0.2 aa substitutions per site.

The result revealed that the sequences of the PRV (Suid herpesvirus 1) in our case has a close connection with the Suid herpesvirus 1 isolate HB1201, Suid herpesvirus 1 strain ADV32751/Italy2014 (*6*), Suid herpesvirus 1 strain TJ (*7*) and Suid herpesvirus 1 isolate HLJ8

(*8*), among which three were observed in China. The Suid herpesvirus 1 strain TJ is an emergent and highly pathogenic PRV variant with unique molecular signatures clustering in one clade with Suid herpesvirus 1 isolate HLJ8. Both strains were isolated from the northern regions of China and were related to the recent years' PRV -like outbreaks occurred in numerous Bartha-K61-vaccinated pig farms in multiple regions in China.

References

1. Ma W, Lager KM, Richt JA, Stoffregen WC, Zhou F, Yoon KJ. Development of real-time polymerase chain reaction assays for rapid detection and differentiation of wild-type pseudorabies and gene-deleted vaccine viruses. J Vet Diagn Invest. 2008;20:440–7. PubMed http://dx.doi.org/10.1177/104063870802000405

2. Wernike K, Beer M, Freuling CM, Klupp B, Mettenleiter TC, Müller T, et al. Molecular double-check strategy for the identification and characterization of Suid herpesvirus 1. J Virol Methods. 2014;209:110–5. PubMed http://dx.doi.org/10.1016/j.jviromet.2014.08.022

3. Nonaka CKV, Fonseca AA Junior, Guedes EO, D'Ambros RM, Lima GK, Camargos MF, et al. Different methods of real-time PCR for detection of pseudorabies virus. Ciência Rural. 2017;47:1–7. http://dx.doi.org/10.1590/0103-8478cr20160342

4. Shen Y, Zhang S, Sun R, Wu T, Qian J. Understanding the interplay between host immunity and Epstein-Barr virus in NPC patients. Emerg Microbes Infect. 2015;4:e20. PubMed http://dx.doi.org/10.1038/emi.2015.20

5. Krech U. Complement-fixing antibodies against cytomegalovirus in different parts of the world. Bull World Health Organ. 1973;49:103–6. PubMed

6. Pizzurro F, Mangone I, Zaccaria G, De Luca E, Malatesta D, Innocenti M, et al. Whole-genome sequence of a Suid herpesvirus-1 strain isolated from the brain of a hunting dog in Italy. Genome Announc. 2016;4:e01333–16. PubMed http://dx.doi.org/10.1128/genomeA.01333-16

7. Luo Y, Li N, Cong X, Wang CH, Du M, Li L, et al. Pathogenicity and genomic characterization of a pseudorabies virus variant isolated from Bartha-K61-vaccinated swine population in China. Vet Microbiol. 2014;174:107–15. PubMed http://dx.doi.org/10.1016/j.vetmic.2014.09.003

8. Ye C, Guo JC, Gao JC, Wang TY, Zhao K, Chang XB, et al. Genomic analyses reveal that partial sequence of an earlier pseudorabies virus in China is originated from a Bartha-vaccine-like strain. Virology. 2016;491:56–63. PubMed http://dx.doi.org/10.1016/j.virol.2016.01.016

Technical Appendix Table 1. Primers used in polymerase chain reaction

Target gene	Primers sequence (5′-3′)	Amplicon (bp)
PRV -gB-718F	ACAAGTTCAAGGCCCACATCTAC	94
PRV -gB-812R	GTCYGTGAAGCGGTTCGTGAT	94
PRV -gB-778F	ACCAACGACACCTACACCAAG	79
PRV -gB-856R	CCTCCTCGACGATGCAGTTG	79
PRV -gE-Sybr-R	GGAGAGACGATGGGGTGAGT	116
PRV -gE-Sybr-F	GACGGATGTGATGTTGCTGA	116

Technical Appendix Table 2. Serologic tests result of PRV, EBV and CMV for the target patient and control group*

Sample	PRV-antibodies	EBV-IgG	CMV-IgG
Target Patient	+	+	+
Control 1	-	+	+
Control 2	-	+	+
Control 3	-	+	+
Control 4	-	+	+
Control 5	-	+	+
Control 6	-	+	+
Control 7	-	+	+
Control 8	-	+	+
Control 9	-	+	+
Control 10	-	+	+
Control 11	-	+	+
Control 12	-	+	+
Control 13	-	+	+
Control 14	-	+	+
Control 15	-	+	+
Control 16	-	+	+
Control 17	-	+	+
Control 18	-	+	+
Control 19	-	-	+
Control 20	-	+	+

*PRV, pseudorabies virus; EBV, Epstein-Barr virus; CMV, cytomegalovirus; +, positive; –, negative.

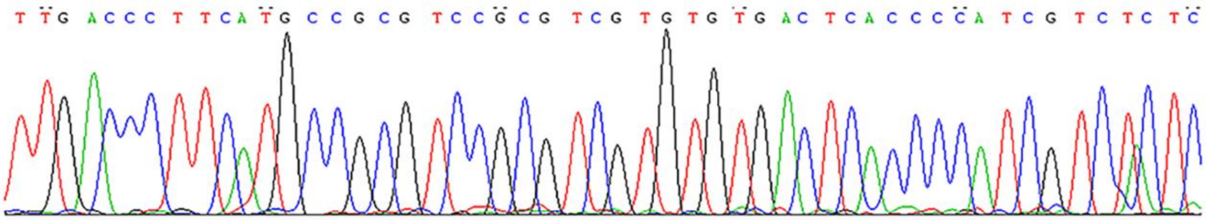

Technical Appendix Figure 1. Sanger sequencing of PCR product identified Suid herpesvirus-1.

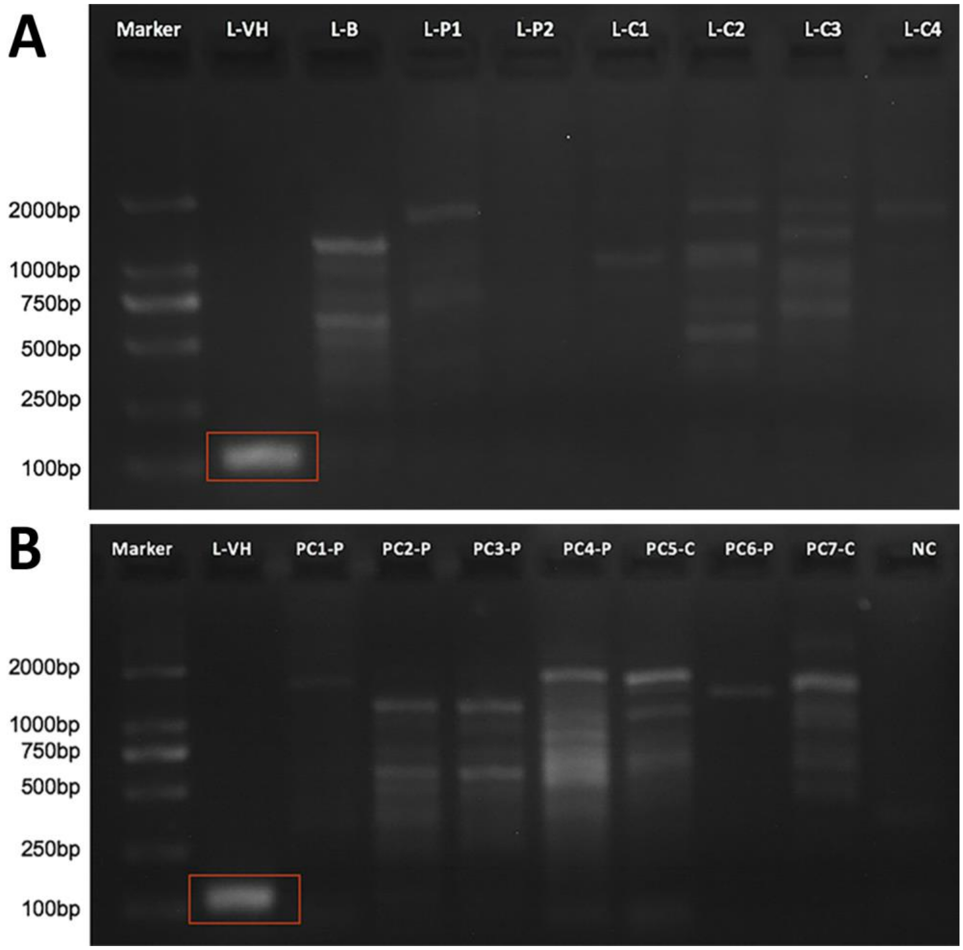

Technical Appendix Figure 2. Electrophoretogram of PCR; the image outlined in red in panels A and B is the PrV amplified product: A) PCR conducted among vitreous humor, plasma, and cerebrospinal fluid obtained from the patient during follow-up exams: L represents this patient, VH represents vitreous humor, B represents blood, P represents plasma, C represents cerebrospinal fluid. B) PCR conducted among samples collected from other patients as control group during the same period: L represents this patient, PC1–7 represents different patient control, VH represents vitreous humor, P represents plasma, C represents cerebrospinal fluid, NC represents negative control.

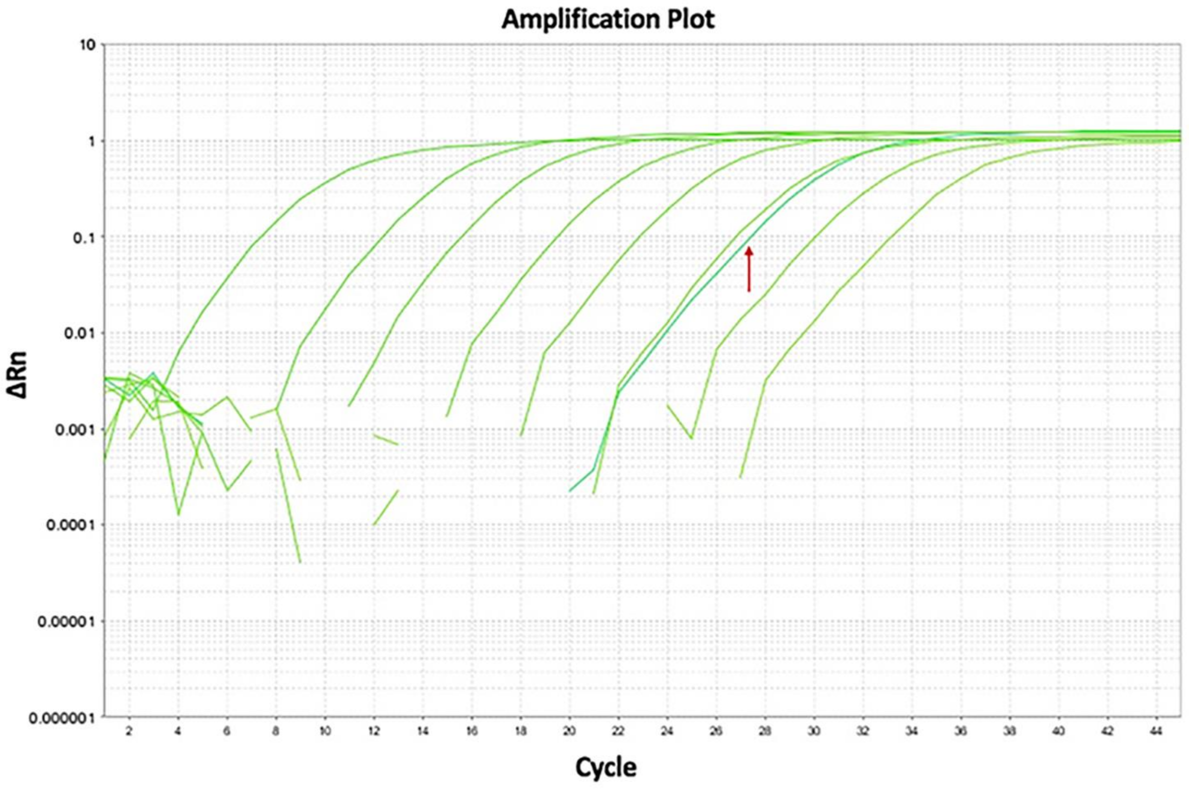

Technical Appendix Figure 3. Amplification curve of the real-time PCR. The dark dotted line with arrow indicates vitreous humor sample; light dotted lines indicate standard curves with different concentration gradients. ΔRn, amount of probe degradation during PCR, which represents the amount of PCR product.

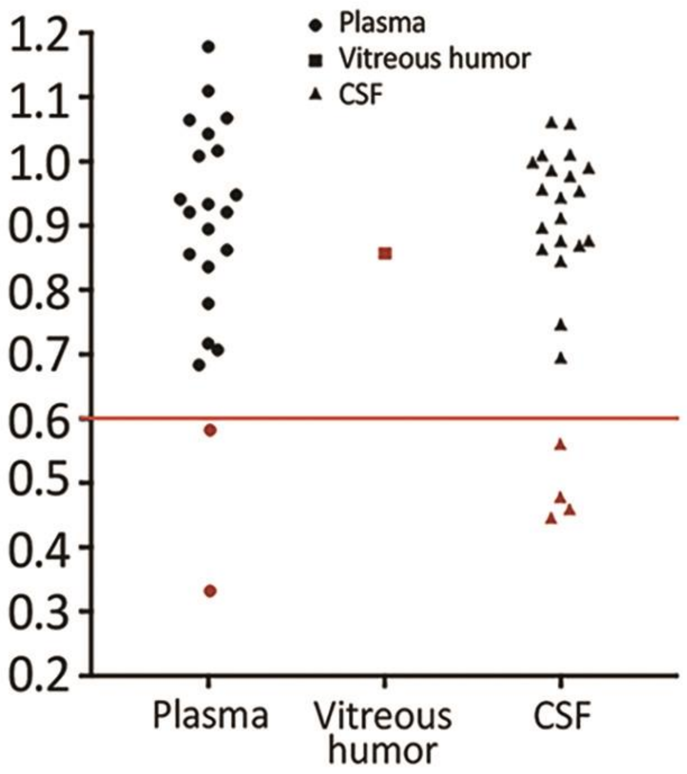

Technical Appendix Figure 4. The result of serologic testing reveals that there were antibodies to pseudorabies virus in the patient's plasma and cerebrospinal fluid. The red dots represent samples from this patient; black dots represent 20 different control samples obtained from other patients randomly chosen from databases during the same period. S/N, sample/negative value; results are considered positive when S/N is $\leq$0.60 (Technical Appendix). The 2 plasma samples collected 4 months and 5 months after disease onset from this patient and the 4 cerebrospinal fluid samples collected during 2 weeks to 2 months after disease onset obtained positive results; the vitreous sample collected during the operation obtained a negative result. All control samples obtained negative results.
